# Non-linear association between mHealth-measured daily steps and subsequent healthcare expenditures among long-term app users in Osaka, Japan

**DOI:** 10.1016/j.ssmph.2026.101958

**Published:** 2026-08-03

**Authors:** Haruka Kato

**Affiliations:** a Department of Living Environment Design, Graduate School of Human Life and Ecology, Osaka Metropolitan University, Osaka, 558-8585, Japan; b Well-being Co-Creation Research Center, Osaka Metropolitan University, Osaka, 536-8550, Japan

**Keywords:** Daily steps, Healthcare expenditures, mHealth, Older adults, Middle-aged adults, Generalized additive models

## Abstract

While physical activity has been associated with lower healthcare expenditures, existing studies relying on self-reports and linear models have struggled to characterize the non-linear shape of this association. Using a linked dataset of mHealth step counts and administrative claims data from 20,950 residents aged ≥40 years, this study employed Generalized Additive Models to elucidate the non-linear association between daily steps and future healthcare expenditures. The outcome was defined as the total healthcare expenditures accrued during the 6 months following each observation month; this 6-month window was chosen a priori for stability and interpretability, not because health effects of walking are assumed to emerge within exactly 6 months. Predicted 6-month healthcare expenditures decreased steeply as daily steps increased from low activity levels and then leveled off at approximately 10,000–12,000 steps/day, indicating a non-linear association between daily steps and subsequent expenditures. This low-expenditure plateau (point-estimate: ∼11,000 steps/day) was associated with a predicted geometric mean expenditure of 17,170 [16,174–18,229] JPY, though confidence intervals widened at higher step counts. Stratified analyses showed similar local nadirs in the data-dense range across age groups despite large differences in absolute magnitudes: the local nadir was 10,612 steps/day (48,407 [45,568–51,423] JPY) for older adults (≥65 years) versus 11,317 steps/day (1514 [1360–1685] JPY) for middle-aged adults, suggesting ∼11,000 steps/day as a common associational benchmark. These associations are observational and cannot establish that increasing walking causally lowers expenditures; rather, the non-linear shape suggests that strategies shifting population activity distributions toward this lower-expenditure range merit further evaluation.

## Highlights

• Large-scale study links mHealth-measured daily steps to healthcare expenditure.
• GAM-predicted 6-month healthcare expenditures level off near 10,000–12,000 steps/day.
• Middle-aged adults show a low-expenditure region with a nadir near 11,000 steps/day.
• Older adults show a similar curve but far higher expenditure levels across steps.
• Findings support hypothesis generation for interventions in low-step populations.

## 1 Introduction

Rapid population aging and the consequent surge in healthcare expenditures pose a critical challenge to the financial sustainability of social security systems worldwide (WHO, 2015). In Japan, national health care expenditures have exceeded 46 trillion JPY (approximately 300 billion USD), accounting for nearly 11% of GDP (MHLWof Japan, 2023). To mitigate this fiscal burden, promoting physical activity (PA) among middle-aged and older adults has been prioritized in global public health policies (Ding et al., 2016; Saito & Podestà, 2024). Healthcare expenditures tend to increase sharply from middle age, driven by the depreciation of health capital and the accumulated burden of non-communicable diseases (NCDs), making this demographic a primary driver of national medical expenditures (Lee et al., 2012). Accordingly, targeting this population represents an essential strategy for preventive investment to extend healthy life expectancy and contain expenditures. Epidemiological evidence consistently links higher PA to reduced mortality and morbidity (del Pozo Cruz, Ahmadi, Lee, et al., 2022; Inoue et al., 2023; Paluch et al., 2022; Saint-Maurice et al., 2020). Moreover, numerous studies have reported that higher PA is associated with lower healthcare expenditures (Nagai et al., 2011; Okamoto et al., 2021; Tsuji et al., 2003). However, the precise relationship between daily PA volume and direct future healthcare expenditures remains understudied, particularly regarding the shape of the association across the range of activity within these high-expenditure, high-risk age groups.

Existing literature predominantly relies on self-reported questionnaires (e.g., the International Physical Activity Questionnaire (IPAQ)), which are prone to recall bias and typically capture PA as categorical variables (Gomes et al., 2020; Kang & Xiang, 2017; Marashi et al., 2019; Tsuji et al., 2003). This categorical approach necessitates linear assumptions, masking specific "tipping points" or saturation effects (Duijvestijn et al., 2023). Without continuous mHealth-derived data, the nuanced "shape" of the economic benefits of walking remains hidden, particularly among middle-aged and older populations, where physiological resilience declines and the economic consequences of physical inactivity are most severe. With the widespread adoption of wrist accelerometer-measured and mobile health (mHealth) devices, it is now possible to collect continuous, objective step count data on a population scale (Kato, 2025; Mönninghoff et al., 2021). This high-resolution data provides a novel opportunity to apply advanced non-linear modeling techniques (del Pozo Cruz, Ahmadi, Lee, et al., 2022; del Pozo Cruz, Ahmadi, Naismith, et al., 2022).

From a health capital and health production perspective, daily walking can be conceptualized as a behavioral input that may contribute to the production and maintenance of health and may therefore be associated with subsequent demand for medical care and healthcare expenditures (Grossman, 1972). However, the marginal productivity of additional steps is unlikely to be constant: increases from very low activity to moderate walking may yield substantial health gains, whereas additional gains are expected to diminish at higher volumes (diminishing marginal returns). Moreover, at very high volumes, walking may entail marginal costs—such as musculoskeletal strain, injury risk, or fatigue—that could increase healthcare utilization for some individuals. Together, diminishing marginal benefits and potentially rising marginal costs imply a non-linear relationship between step counts and future expenditures, suggesting a range over which the association with expenditures flattens rather than an unbounded 'more is better' pattern. Building on this theoretical expectation and the availability of continuous mHealth step data, an important research question arises: What is the precise non-linear trajectory of the relationship between daily steps and future healthcare expenditures among middle-aged and older adults?

The objective of this study is to elucidate the non-linear association between mHealth-measured daily steps and subsequent healthcare expenditures. Based on this non-linear relationship, this study aims to characterize step-count levels associated with lower predicted healthcare expenditures within the analytic cohort. In addition, this study conducts age-stratified analyses to examine whether the fitted step–expenditure trajectories and local nadirs differ between middle-aged adults (40–64 years) and older adults (≥65 years), recognizing their distinct lifestyle patterns and health risks. To achieve these objectives, this study leveraged a large-scale linked dataset of 20,950 long-term A-Smile users aged 40 years or older in Osaka, Japan. Furthermore, this study employed Generalized Additive Models (GAM) to (1) describe associations across alternative future expenditure accumulation windows and (2) estimate non-linear step–expenditure curves and their fitted minima. These analyses were intended to generate hypotheses within this selected cohort rather than establish population-wide step-count targets or intervention effects.

## 2 Materials and Methods

### 2.1 Research design

This study employed a retrospective longitudinal cohort design to examine the non-linear association between objective physical activity and healthcare expenditures. The overall observation period spanned 48 months, from January 2020 to December 2023. Within this timeframe, the primary analysis period was defined as 36 months, from July 2020 to June 2023, to ensure sufficient data availability for both baseline history and future expenditure accumulation (i.e., a complete 6-month baseline window and up to a 6-month future accumulation window for each index month). The study field was Osaka Prefecture, Japan. Osaka Prefecture can be classified as a "super-aged society." As of 2023, the proportion of the population aged 65 and over in Osaka Prefecture was approximately 29% (Osaka Prefecture, 2024), exceeding the World Health Organization's threshold of 21% for a super-aged society (WHO, 2015). This demographic context characterizes the study setting, but the analytic cohort was not designed to represent the entire Osaka population or other aging societies.

The primary predictor was daily step count, acquired through the mHealth application “A-Smile” (Osaka Prefecture's official health promotion platform). The application automatically retrieves step-count data from pedometer sensors embedded in users' smartphones (via Google Fit or Apple HealthKit) or from linked wearable devices. Unlike self-reported physical activity, which is subject to recall bias, this method provides continuous, objective measurement. For this analysis, this study calculated the monthly mean of daily step counts for each user to represent their habitual physical activity level. Previous large-scale studies of smartphone-measured steps have excluded days with fewer than 500–1000 steps as likely reflecting incomplete device carriage (Althoff et al., 2017). In the present study, no such day-level exclusion was applied prior to computing monthly means. Because the exposure was defined as the monthly mean of daily steps, transient low-step days arising from incomplete carriage or measurement error are diluted in the aggregated measure; the potential influence of remaining low-step records on the left tail of the fitted curve is addressed in the Discussion, and interpretation is confined to the data-dense range.

The primary outcome was healthcare expenditures derived from administrative claims data (National Health Insurance and Medical Care System for the Elderly). The "healthcare expenditures" in this study were defined as gross total healthcare expenditures, meaning 100% of the expenditure, including both the insurer's coverage and the patient's copayment. This comprehensive sum covers inpatient care, outpatient visits, dental services, and pharmacy dispensing expenditures. Expenditures related to uninsured treatments were excluded. It should be noted that only total healthcare expenditure data were available due to privacy protection policies; no data on healthcare expenditure by disease category was available.

To examine the association between step counts and healthcare expenditures independent of potential confounders, this study adjusted for the following covariates, selected for their clinical and economic relevance. Regarding demographic factors, age (continuous) and gender (male/female/other) were obtained from the mHealth app registration data linked to the resident registry. Body Mass Index (BMI) was obtained from self-reported data entered by users via the mHealth application. Self-reported BMI was assigned at the individual level (each user's mean of recorded BMI). It was missing for 5376 of 20,950 individuals (25.7%), who had no BMI entry in any month. Missing values had initially been stored under a zero code; we identified and corrected this during revision. In the primary model, missing BMI was imputed using the observed median (22.7); its influence on the step curve was assessed by re-estimating the model with the BMI smooth omitted (Section 3.5). A binary variable for health checkup attendance (Yes/No in each month) was also included to control for health consciousness and access to preventive care. To account for potential confounding by underlying health status—where ill health leads to both lower physical activity and higher expenditures—this study adjusted for "baseline medical expenditures," defined as the cumulative expenditures incurred in the 6 months preceding the observation point. This serves as a robust proxy for the user's underlying health status and chronic disease burden. Given the study period (2020–2023), a binary variable indicating the months during which a state of emergency was declared in Osaka Prefecture was included to control for period effects on both physical activity and healthcare-seeking behaviors. Because BMI may act as either a confounder or a mediator in the relationship between physical activity and healthcare expenditures, we conducted a sensitivity analysis that re-estimated the primary GAM on the same analytic observations after omitting the BMI smooth term. This analysis assessed whether conditioning on BMI materially changed the estimated step–expenditure trajectory.

In defining the dependent variable, this study addressed two key temporal dynamics. First, previous epidemiological research emphasizes that the impact of environmental and behavioral factors on health outcomes and associated expenditures is often not immediate but manifests with a time delay (lagged effects) (Josa-Culleré et al., 2024). Second, from a health services perspective, patients with stable chronic conditions often visit medical institutions only once every two to three months rather than monthly. Consequently, a strictly monthly analysis might overlook expenditures incurred in adjacent months, failing to capture the true economic burden of chronic diseases. To capture typical utilization cycles while acknowledging potentially delayed effects, we evaluated alternative future expenditure accumulation windows (k = 1 to 6 months after the index month). Because these candidate models correspond to different outcome definitions (i.e., different accumulation windows), AIC/GCV values are reported descriptively and were not interpreted as formal evidence of a uniquely “optimal” causal delay. We selected the 6-month accumulation window as the primary outcome horizon a priori for interpretability and stability, and we report results for other windows as sensitivity analyses.

Finally, to investigate age-specific step–expenditure trajectories, this study performed stratified analyses by grouping the dataset into two groups: middle-aged and older adults. Separate GAMs were fitted for each age group to identify distinct non-linear trajectories and expenditure-minimization points appropriate for each life stage.

### 2.2 Data

Step-count data were collected from "A-Smile," an official mHealth app for Osaka residents aged 18 and over (Osaka Prefecture, 2025a). Provided by the Osaka Prefectural Government, the app automatically records health behaviors, including step counts via smartphone sensors (Google Fit or Apple HealthKit) or linked wearables. A-Smile is promoted by the prefecture through prefectural and municipal public relations, registration support at local health checkup venues, and workplace wellness campaigns. Enrollment is voluntary: any eligible resident may install the app of their own accord. Recruitment, therefore, relies on self-selection rather than probability sampling, a point relevant to the representativeness of the source data. For the present analysis, the study population was restricted to users aged 40 years and older. Furthermore, to evaluate economic outcomes, this study used a linked dataset that integrates app-derived step counts with administrative claims data. Because claims data were available exclusively for beneficiaries of the National Health Insurance and the Medical Care System for the Elderly, the final analytic sample consisted of individuals enrolled in these specific insurance schemes for whom both objective physical activity records and healthcare expenditure data were successfully linked. For this study, data on step count and personal attributes, including gender, age, and BMI, were used.

Prefectural Government has positioned the secondary use of this large accumulated dataset to inform health-promotion measures and healthcare expenditure optimization policies. Therefore, upon registration, the app-users provide comprehensive consent to the secondary use of their data in a form that does not personally identify them (Osaka Prefecture, 2025c, 2025d). This distinguishes it from purely commercial apps and may be viewed as a public-sector mHealth platform that enables the use of secondary data for policy evaluation, subject to user consent and data governance. Based on this data usage policy, academic research using A-Smile has been published and has begun to inform local public health practice and policy discussions (Koyama et al., 2021; Oyama et al., 2025; Yokoyama & Kitano, 2024). This study also used these research data with the permission and cooperation of Osaka Prefecture, as in the projects.

### 2.3 Statistical analysis

We employed Generalized Additive Models (GAM) to capture complex, non-linear associations without imposing a priori functional forms (Wood, 2017). Unlike linear models used in previous studies (Kang & Xiang, 2017; Marashi et al., 2019; Nagai et al., 2011; Tsuji et al., 2003), GAM replaces linear predictors with penalized regression splines, enabling flexible estimation of associations from continuous step-count data. To contextualize model fit and isolate the contribution of the non-linear step term, we additionally fitted three comparator specifications using the same analytic observations and 6-month outcome: an intercept-only null model; a fully linear model in which steps, age, BMI, and baseline medical expenditures were entered as linear terms; and a step-linear model that retained smooth terms for age, BMI, and baseline medical expenditures but entered daily steps as a linear term. Deviance and AIC were compared across the four specifications.

The unit of analysis was the person-month. Because multiple months were observed per individual and future cumulative expenditure windows overlap across adjacent index months, we accounted for within-person dependence when constructing uncertainty intervals by using an individual-level resampling (cluster bootstrap) for the predicted curves and key derived quantities (e.g., the local minimum). Let i denote individuals and t denote calendar months. Monthly physical activity ($Steps_{i,t}$) was defined as the monthly mean of daily step counts. For each candidate lag length k∈{1,2,3,4,5,6} months, the outcome was defined as the cumulative healthcare expenditures accrued during the subsequent k months (that is, excluding the index month t), as defined in Eq. (1):

(1) $$Y_{i,t}^{k}=\sum_{h=1}^{k}\mathrm{Cos}t_{i,t+h}$$

This construction reflects the hypothesized delayed economic impact of physical activity and aligns with typical healthcare utilization patterns that may not occur strictly monthly.

To mitigate reverse causality (that is, poor health leading to both lower steps and higher expenditures), this study adjusted for baseline medical expenditures defined as the cumulative expenditures during the prior 6 months, as defined in Eq. (2):

(2) $$BaselineCost_{i,t}^{\left(6\right)}=\sum_{h=1}^{6}\mathrm{Cos}t_{i,t-h}\text{.}$$

Person-months lacking a complete expenditure history for the baseline window or a complete follow-up for the evaluated lag window were excluded from the corresponding analyses. Because healthcare expenditures include many zeros and are right-skewed, the dependent variable was log-transformed as $\log\left(Y_{i,t}^{k}+1\right)$ using the natural logarithm.

For each candidate lag k, this study fitted a Gaussian GAM (identity link) using the following additive structure in Eq. (3):

(3) $$\log\left(Y_{i,t}^{k}+1\right)=\alpha +s_{1}\left(Steps_{i,t}\right)+s_{2}\left(Age_{i,t}\right)+s_{3}\left(BMI_{i,t}\right)+s_{4}\left(BaselineCost_{i,t}^{\left(6\right)}\right)+\beta^{⊤}X_{i,t}+\varepsilon_{i,t}$$

where $s_{m}\left(\cdot \right)$ are penalized regression spline smooth functions, and $\beta^{⊤}$ $X_{i,t}$ represents the linear component of the model for categorical covariates. Specifically, $X_{i,t}$ includes gender (male/female/other), monthly health checkup attendance (yes/no), and a binary indicator for whether the month fell within a COVID-19 emergency declaration period, and β is the corresponding vector of coefficients. In implementation, the step-count smooth was specified with 10 spline basis functions (n_splines = 10), chosen a priori as a moderate basis dimension expected to capture plausible curvature in the step–expenditure relationship while limiting overfitting, with its adequacy confirmed post hoc by the n_splines sensitivity analysis (Section 3.5, Table 8), while other smooth terms used the package defaults. To evaluate the complexity of the non-linear associations, the Effective Degrees of Freedom (EDoF) was calculated for each smooth term. An EDoF value of 1.0 indicates a linear relationship, while values significantly greater than 1.0 indicate a non-linear relationship. Missing data were handled as follows. Because administrative claims record only reimbursed services, the absence of a claim in a covered person-month was treated as a true zero—indicating that no insured care was used in that month—rather than as unobserved data; as described above, person-months lacking a complete baseline or follow-up window were excluded from the corresponding analyses. This zero-coding rule was applied only to covered person-months with linkable claims data; individuals without linkable healthcare expenditure data were excluded from the analytic sample rather than coded as zero. Self-reported BMI was missing for 5376 of 20,950 individuals (25.7%), who recorded no BMI in any month; missing values had been stored under a zero code in the source file, which we identified and corrected during revision. In the primary model these were imputed using the observed median (22.7); sensitivity to this choice was assessed by re-estimating the model with the BMI smooth omitted (Section 3.5). Categorical covariates were encoded as factors.

To identify the temporal delay between physical activity and healthcare expenditures, this study estimated models for k=1 to 6 months and examined AIC and GCV across windows. As noted above, because these correspond to different outcome definitions, these criteria were used descriptively rather than to identify a uniquely optimal causal delay; the 6-month window was adopted a priori as the primary horizon.

For the primary 6-month outcome horizon, this study visualized the modeled association between steps and future healthcare expenditures by generating predictions across a grid of step counts, holding other covariates constant at typical values (median for continuous covariates and mode for categorical covariates). For presentation, the step-count axis was restricted to the range from 0 up to the 99.5th percentile of observed steps to reduce the visual influence of extreme values.

Predicted values were obtained on the log scale and then back-transformed to the original expenditure scale using exp⁡(·)−1. Pointwise 95% confidence intervals were computed on the log scale using the fitted GAM and back-transformed for presentation. To characterize potential non-linear minimum, this study identified (i) the global minimum of the predicted expenditure curve within the plotted step range and (ii) local minima when present (that is, multiple expenditure-minimizing regions), based on the discretized prediction grid. A rug plot was additionally included to display the empirical density of observed step values along the x-axis. Here, the “global minimum” denotes the lowest predicted expenditure across the entire modeled step-count range used for plotting (i.e., from 0 to the 99.5th percentile of observed steps). The “local nadir” refers to an expenditure-minimizing point within a practically attainable range where observations are more densely concentrated, and is therefore emphasized for interpretation because it lies within the data-dense range rather than the sparse, extrapolated tail.

To visually summarize how predicted expenditures vary across combinations of step counts and age, this study constructed a 3D prediction surface from the fitted additive GAM. This study generated a two-dimensional mesh-grid spanning steps from 0 to the 99.5th percentile and age from the minimum to maximum observed values, computed predicted expenditures at each grid point, and held all other covariates constant at their median or mode values (gender, BMI, health checkup attendance, baseline expenditure, and COVID-19 indicator). Because the model is additive (that is, it does not include a steps-by-age interaction smooth), the 3D surface represents model-based predictions under the additive specification rather than an explicitly estimated bivariate interaction.

To investigate age-specific associations and descriptive lower-expenditure step-count ranges, this study conducted stratified analyses, fitting the GAM specification separately for middle-aged and older adults, and repeated the lag selection and prediction-based identification of expenditure-minimizing step counts within each subgroup.

All statistical analyses were performed in Python (version 3.12.13) using Google Colaboratory, with GAMs fitted using the pyGAM package (version 0.12.0).

To assess the robustness of the estimated step–expenditure curve, we conducted additional sensitivity analyses on the primary 6-month specification. First, to evaluate dependence on the smoothing basis, the step smooth was re-estimated with n_splines = 5, 8, 10, 15, 20, and 25. Second, regarding missing covariate data, BMI was missing for 25.7% of individuals (47.8% of analytic person-months) and was median-imputed in the primary model; we assessed sensitivity to this choice by re-estimating the step curve with the BMI smooth omitted entirely, which removes any dependence on the imputed values. Third, to address the COVID-19 exposure period, the model was re-estimated after (i) excluding months under a state-of-emergency declaration and (ii) excluding calendar years 2020–2021. The omission of the BMI smooth term and the comparator (null, fully linear, and step-linear) specifications are described above. Results of all sensitivity analyses are reported in Section 3.5.

### Ethical statement

The research protocol was approved by the Ethics Committee of the Graduate School of Human Life and Ecology, Osaka Metropolitan University (No.24-42). Informed consent was obtained electronically from all users, and participation could be declined at any time through the smartphone application, in accordance with the Declaration of Helsinki (Osaka Prefecture, 2025c, 2025d). Data processing complied with Japan's Next-Generation Medical Infrastructure Act, ensuring anonymity through government-accredited businesses (Cabinet Office of Japan, 2025; Osaka Prefecture, 2025d).

## 3 Results

### 3.1 Sample characteristics

**Fig. 1:**
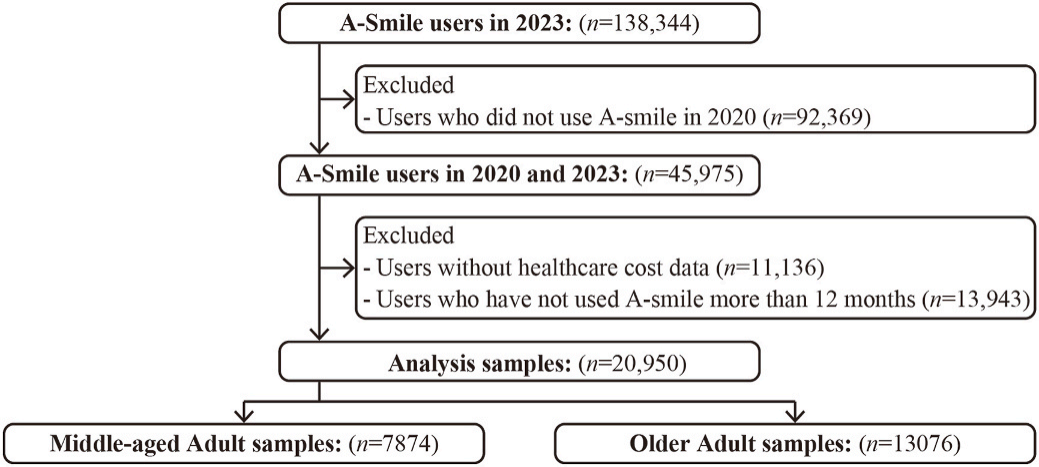
**Flowchart of study population selection.** The diagram illustrates the process of excluding users from the initial user base of the 'A-Smile' mHealth application.

Fig. 1 illustrates the study population selection process. The initial dataset comprised 138,344 users registered with the A-Smile application as of 2023. This study excluded 92,369 users who did not use the app in 2020. Of the remaining 45,975 users, this study excluded 25,025 individuals who met at least one exclusion criterion: missing healthcare expenditure data (n = 11,136) and/or insufficient app usage (less than 12 months; n = 13,943). Fifty-four individuals met both criteria. Consequently, the final analytic sample consisted of 20,950 individuals. This corresponds to a retention of 15.1% (20,950/138,344) of the registered base, confirming that the analytic cohort is a highly selected group of users who both sustained app engagement and were enrolled in the National Health Insurance or the Medical Care System for the Elderly with linkable claims.

Table 1 presents stratified baseline characteristics. Older adults (62.4%) had significantly higher mean daily steps and monthly healthcare expenditures than middle-aged adults (p < 0.01). Mean daily steps were 5363 among older adults and 5092 among middle-aged adults, an absolute difference of 271 steps/day. Females were overrepresented in the middle-aged group (67.7%), while older adults had slightly higher BMI. The slightly higher mean steps among older than middle-aged users—counterintuitive for an age gradient—are consistent with stronger health and digital selection among older app users rather than an age effect, as quantified below.

**Table 1:** Sample characteristics.

	unit	Middle-aged adults	Older Adults	*p-*value
**Observation**	n	7874	13,076	
**Daily Steps:**	Means (SD)	5092 (3217)	5363 (3327)	< 0.01
**Monthly Expenditure:**	Means (SD)	20,210 (52,337)	31,346 (62,450)	< 0.01
**BMI:**	Means (SD)	23.76 (6.84)	24.05 (6.77)	< 0.01
BMI Missing:	n (%)	2393 (30.4%)	2983 (22.8%)	
**Age (Years)**	Means (SD)	55.19 (6.45)	70.71 (3.31)	< 0.01
**Gender**				
Male:	n (%)	2542 (32.3%)	6165 (47.1%)	< 0.01
Female:	n (%)	5330 (67.7%)	6911 (52.9%)	
Other:	n (%)	2 (0.0%)	0 (0.0%)	

**Note:** BMI means (SD) are computed among individuals with at least one recorded BMI. "BMI Missing" denotes individuals with no BMI entry in any month (zero-coded in the source file); these were median-imputed in the primary model and assessed by a BMI-omitted sensitivity specification (Section 3.5).

To describe observable differences across cohort selection stages, Table 2 compares daily steps and sex across the registered base, sustained users, and the linked analytic sample. Restricting the registered base to sustained users (active in both 2020 and 2023) was associated with higher mean steps (6053 to 6727 steps/day), consistent with engagement-related selection, in which more active users are more likely to sustain app use. In the opposite direction, restricting this pool to enrollees in the National Health Insurance or the Medical Care System for the Elderly with linkable claims lowered the mean to 5261 steps/day; the 25,025 users excluded at this stage—disproportionately younger, commuting-age individuals without linkable public-insurance claims—were correspondingly more active (Table 2). Selection thus operated in two opposing directions, yielding an analytic sample skewed toward the insured, less ambulatory segment of sustained users. These comparisons quantify selection in observed step counts and sex composition, but they do not estimate the magnitude of selection bias in the fitted expenditure curve because healthcare expenditures were not linkable for excluded users and the source population was not probability-sampled. The fitted curve should therefore not be generalized to all middle-aged and older residents of Osaka.

**Table 2:** Representativeness of the analytic sample across cohort selection stages.

	unit	A-Smile users 2023	A-Smile users in 2020 and 2023	Analysis sample
Observation:	n	138,344	45,975	20,950
Daily steps in 2023:	Means	6053 (6451)	6727 (9568)	5261 (3288)
Gender: Female:	n (%)	74,075 (53.5%)	26,689 (58.1%)	12,241 (58.4%)

**Note:** Daily steps and sex distribution are compared across the A-Smile registered base (2023), sustained users (active in both 2020 and 2023), and the linked analytic sample.

To examine the characteristics of our sample relative to the general population, we referenced publicly available statistics from Osaka Prefecture as of December 2023 (Osaka Prefecture, 2025b). For healthcare expenditures, daily step counts, and BMI, comparable aggregated data for Osaka Prefecture were not publicly available. We analyzed the gender distribution and estimated the mean age of the general population using weighted averages derived from published 5-year age-group data. The analysis revealed distinct demographic discrepancies between the general population and our study sample. In the general population of Osaka, the mean age was 49.6 years for middle-aged adults and 75.0 years for older adults. Furthermore, the proportion of females was 49.9% in the middle-aged group and 56.8% in the older adult group. In contrast, our study sample exhibited a unique demographic profile: middle-aged users were older than the general average (mean 55.2 years), whereas older users were notably younger (mean 70.7 years). Regarding gender, females were substantially overrepresented in the middle-aged sample (67.7%), while the proportion of females in the older sample (52.9%) was lower than the general population benchmark. This pattern suggests that the mHealth app primarily attracts health-conscious women in their 50s and "younger" older adults capable of using smartphones, while underrepresenting the oldest-old (e.g., ≥80 years) who are more likely to be institutionalized or bedridden.

### 3.2 GAM result

Table 3 reports model diagnostics across alternative accumulation windows (k = 1–6 months). We present 6-month cumulative healthcare expenditures as the primary outcome horizon, motivated by typical chronic-care utilization cycles and estimation stability; results for shorter horizons are provided as sensitivity analyses.

**Table 3:** Descriptive comparison of alternative future expenditure accumulation windows.

Lag (Months)	AIC	GCV
1:	1,910,734	16.50
2:	1,858,261	14.13
3:	1,831,450	13.06
4:	1,821,032	12.66
5:	1,812,817	12.36
6:	1,805,557	12.10

**Note:** AIC denotes "Akaike Information Criterion." GCV denotes "Generalized Cross-Validation."

Table 4 summarizes the results of the primary GAM specification. All included predictors—daily steps, age, gender, BMI, health checkup attendance, past medical expenditures, and the COVID-19 period—were statistically significant (p < 0.01). The EDoF indicated the complex, non-linear nature of the associations. Daily steps exhibited a highly nonlinear relationship with healthcare expenditures (EDoF = 9.0), with a non-monotonic pattern in which predicted expenditures were higher in the low-step range and showed unstable behavior in the sparse high-step range. The final model explained 32.6% of the deviance in future healthcare expenditures. This level of explained deviance suggests that the semi-parametric GAM approach may capture complex, non-linear associations in medical costs more flexibly than rigid linear assumptions.

**Table 4:** Results of generalized additive model (GAM).

Variable	Type	EDoF	*p*-value
Steps:	Smooth	9.0	< 0.01
Age:	Smooth	18.2	< 0.01
Gender:	Factor	1.0	< 0.01
BMI:	Smooth	15.6	< 0.01
Health Checkup:	Factor	1.0	< 0.01
Past Expenditure:	Smooth	10.3	< 0.01
COVID-19 Period:	Factor	1.0	< 0.01
**Model Fit**			
Pseudo R^2^:	0.326		
AIC:	1,805,557		

**Note:** EDoF denotes "Effective Degrees of Freedom." AIC denotes "Akaike Information Criterion."

**Fig. 2:**
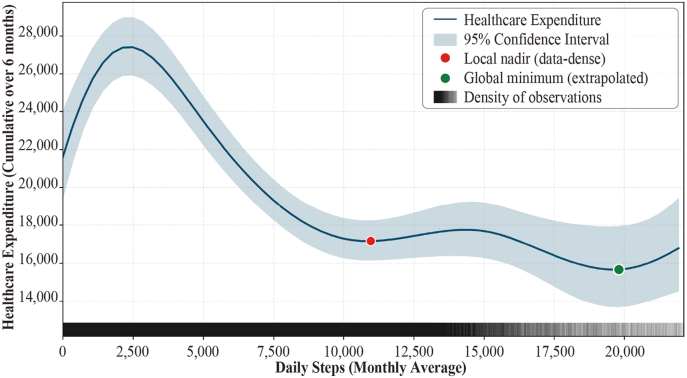
**Non-linear association between daily step count and cumulative healthcare expenditures.** The solid blue line represents the predicted 6-month cumulative healthcare expenditures derived from GAM. The shaded area indicates 95% confidence interval. The red circle marks the local nadir (approximately 11,000 steps), within the data-dense range, beyond which predicted expenditure levels off. The green circle marks the numerical global minimum (approximately 20,000 steps), which lies in a sparse, extrapolated region and is not interpreted as a target. The rug plot along the x-axis depicts the density of participant observations.

Based on this primary model, Fig. 2 describes how predicted healthcare expenditures vary across the range of observed step counts. The analysis identified a local nadir in the expenditure curve at approximately 11,000 steps/day (point estimate: 10,893 steps/day). The predicted geometric mean of cumulative expenditure at this nadir was 17,170 [95% CI: 16,174–18,229] JPY. It should be noted that these values represent the geometric mean derived from log-transformed data, which is lower than the arithmetic mean (total sums) due to the right-skewed distribution of healthcare costs. The model identified a numerical global minimum at a higher intensity of 19,674 steps/day (15,659 [95% CI: 13,693–17,922] JPY). However, this point lies in a data-sparse region (as shown in the rug plot) with wide confidence intervals.

To formally test whether these two points were distinguishable, we compared predicted expenditures at the local nadir (10,893 steps/day) and the numerical global minimum (19,674 steps/day) using an individual-level cluster bootstrap (B = 500). The difference was not statistically distinguishable from zero (local − global = 1048 [95% CI: −4892 to 7006] JPY; ratio 1.09 [95% CI: 0.78–1.54]). This indicates that predicted expenditures across the ∼11,000-to-20,000-step range cannot be reliably differentiated. Consistent with this, the 95% confidence intervals overlap across the 10,000–12,000 step/day range, indicating a broad low-expenditure zone rather than a sharp minimum. As defined in the Methods, the global minimum is the lowest point within the modeled step range, whereas the local nadir represents a more practically relevant minimum within the empirically dense step range. Taken together, these results indicate that predicted expenditures level off once the ∼11,000 step/day region is reached and remain statistically indistinguishable thereafter, rather than identifying a single expenditure-minimizing step count.

### 3.3 Subgroup analysis

To elucidate generation-specific characteristics, this study performed stratified analyses for middle-aged adults and older adults. First, with respect to the descriptive comparison of future expenditure accumulation windows, results were consistent across age groups. As shown in Table 5, the AIC and GCV values were lowest at the 6-month lag for both middle-aged and older adults. These results suggest that a medium-term cumulative outcome definition may be useful for describing step–expenditure associations across age groups, but they should not be interpreted as identifying the causal timing of economic benefits from physical activity.

**Table 5:** Descriptive comparison of alternative future expenditure accumulation windows by age group.

	Middle-aged adults		Older adults	
Lag (Months)	AIC	GCV	AIC	GCV
1:	675,472	17.82	1,221,926	14.91
2:	674,079	17.61	1,160,590	11.29
3:	671,322	17.20	1,132,023	9.92
4:	670,119	17.03	1,122,056	9.48
5:	668,794	16.84	1,114,986	9.18
6:	667,274	16.62	1,109,410	8.95

**Note:** AIC denotes "Akaike Information Criterion." GCV denotes "Generalized Cross-Validation."

Table 6 compares the GAM results between the two groups. A marked difference in the model's explanatory power was observed. The model for older adults explained 41.80% of the deviance in healthcare expenditures, whereas the model for middle-aged adults explained only 22.7%. This disparity suggests that healthcare utilization among older adults is more strongly associated with daily physical activity and physiological factors captured in the model. Conversely, the lower explanatory power among middle-aged adults suggests that their medical expenditures may be driven by a broader array of unobserved heterogeneity, such as occupational factors or lifestyle determinants less correlated with routine step counts. Specifically, daily step counts exhibited a highly nonlinear relationship (EDoF = 8.7 in middle-aged adults and 8.9 in older adults) in both groups.

**Table 6:** Comparison of GAM results between Age groups.

		Middle-aged adults		Older adults	
	Type	EDoF	*p*-value	EDoF	*p*-value
Steps:	Smooth	8.7	< 0.01	8.9	< 0.01
Age:	Smooth	18.1	< 0.01	14.0	< 0.01
Gender:	Factor	1.0	0.329	1.0	< 0.01
BMI:	Smooth	14.5	< 0.01	15.3	< 0.01
Health Checkup:	Factor	1.0	< 0.01	1.0	< 0.01
Past Expenditure:	Smooth	8.4	< 0.01	13.0	< 0.01
COVID-19 Period:	Factor	1.0	< 0.01	1.0	< 0.01
**Model Fit**					
Pseudo R^2^:		0.227		0.418	
AIC:		667,274		1,109,410	

**Note:** EDoF denotes "Effective Degrees of Freedom." AIC denotes "Akaike Information Criterion."

**Fig. 3:**
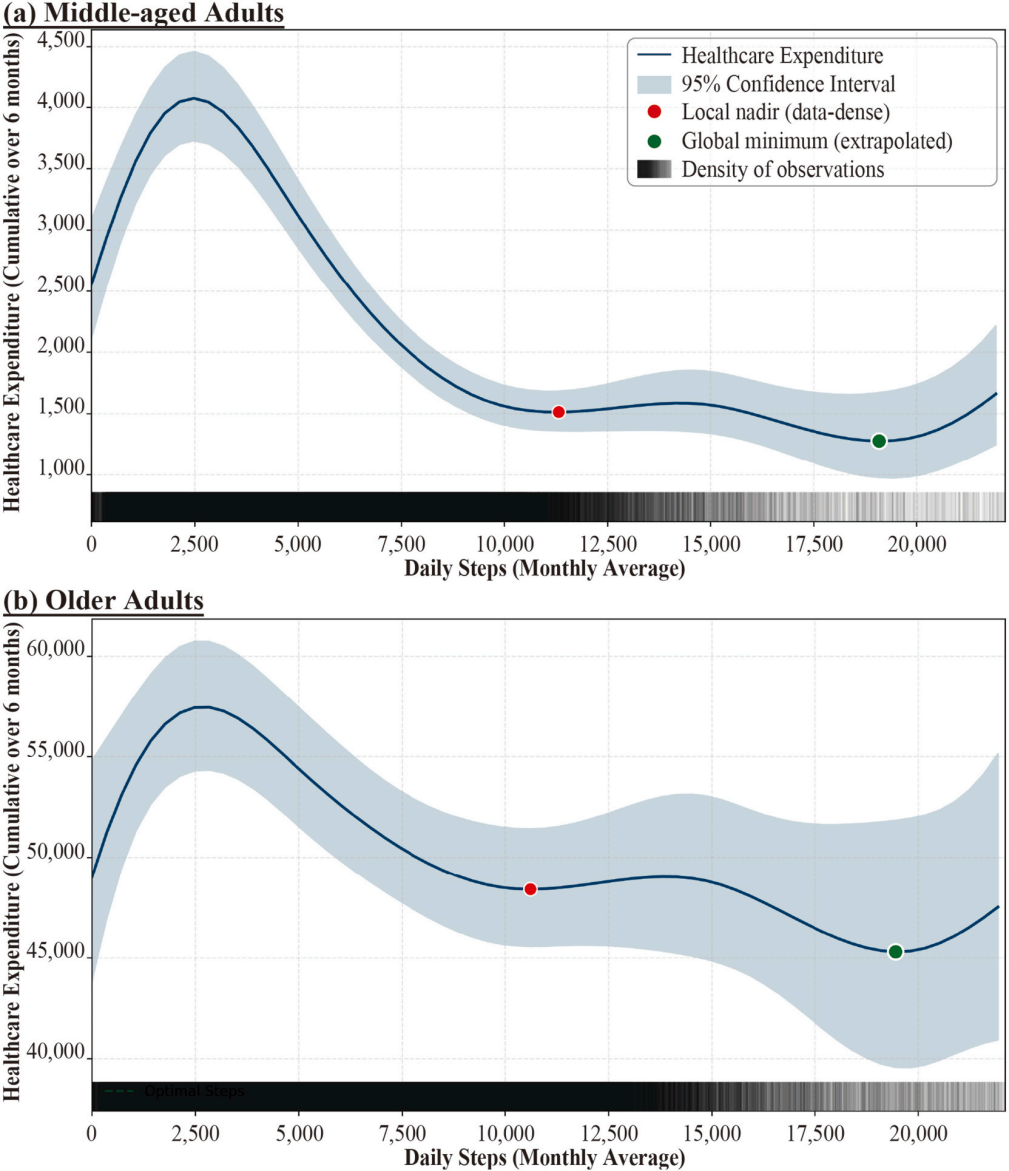
**Stratified non-linear associations by age group. (a)** Middle-aged adults; **(b)** Older adults. The solid lines represent the predicted trajectories of healthcare expenditures. Note the difference in the scale of the y-axis between the two panels, reflecting the higher baseline expenditures in the older adult population. Similar to the overall model, red circles indicate the data-dense local nadir, and green circles indicate the numerical global minimum in the sparse, extrapolated high-step region. The non-monotonic pattern is consistent across generations, although the absolute economic impact is substantially greater among older adults. Shaded areas represent 95% confidence intervals.

Fig. 3 shows that the stratified analysis revealed distinct local nadirs in the data-dense range for each life stage, while the non-monotonic trend remained consistent. For middle-aged adults, the lowest healthcare expenditures (local nadir) were observed at 11,317 steps/day, corresponding to 1514 [95% CI: 1360–1685] JPY. This relatively low predicted value, compared to the arithmetic mean shown in Table 1, reflects the log-normal model's sensitivity to the high prevalence of zero or low-expenditure months among the healthier middle-aged population. A numerical global minimum was observed at 19,096 steps/day (1278 [95% CI: 976–1673] JPY), but—as in the overall model—this point lies in the sparse, extrapolated high-step region and is not interpreted as a target.

In contrast, for older adults, a steep reduction in expenditures was observed up to a local nadir of 10,612 steps/day, corresponding to 48,407 [95% CI: 45,568–51,423] JPY. Similar to the middle-aged group, a global minimum was identified at 19,455 steps/day (45,297 [95% CI: 39,576–51,846] JPY) in the same sparse, extrapolated region. Notably, while the data-dense local nadir (approximately 11,000 steps/day) and the overall trajectory shape were stable across age groups, the absolute magnitude of predicted expenditures was substantially higher in older adults. This likely reflects the age-related increase in baseline medical needs, even among those who maintain relatively high levels of physical activity. The finding that the local nadir occurs at approximately 11,000 steps/day (10,600–11,300) for both age groups suggests a common descriptive benchmark for the lower-expenditure portion of the fitted association in this selected cohort.

### 3.4 Verification of non-linear association

**Fig. 4:**
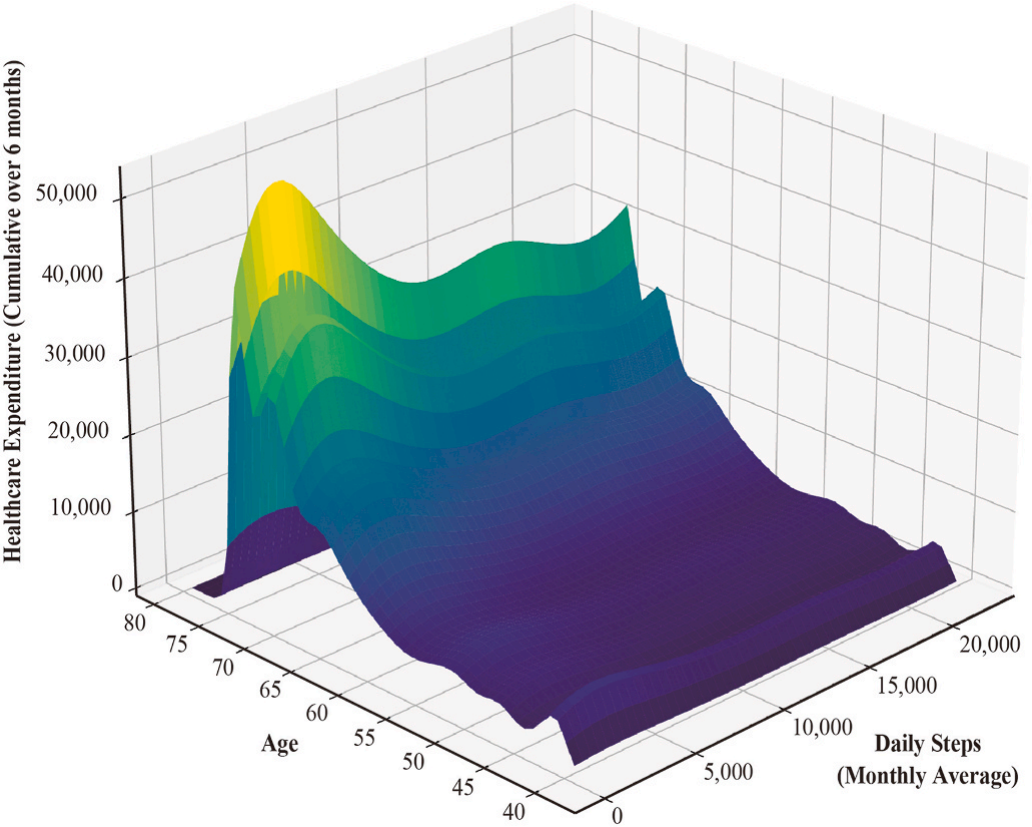
**Three-dimensional surface plot of predicted healthcare expenditures by age and daily steps.** The vertical axis and color gradient represent the predicted 6-month cumulative healthcare expenditures, with yellow indicating higher expenditures and dark blue indicating lower expenditures. Because the fitted GAM is additive and does not include a steps-by-age interaction smooth, this plot visualizes model-based predictions across age and step values rather than a formally estimated interaction. The steep slope descending from the "high-age/low-step" peak (yellow region) indicates that the adjusted association between steps and predicted expenditures is steepest among older, low-step observations. Other covariates (gender, BMI, baseline expenditures, etc.) were held constant at their respective medians or modes for this visualization.

Finally, Fig. 4 presents a 3D surface plot visualizing the joint interaction between age and daily steps. The yellow peak represents the highest predicted expenditures, clustering in the "high-age/low-step" region. Because the fitted GAM is additive and does not include a steps-by-age interaction smooth, this visualization should not be interpreted as evidence of a formally estimated statistical interaction. The distinct "cliff-like" slope descending from this peak indicates that the adjusted step–expenditure association is steepest among older, low-step observations. This steep gradient levels off significantly as it approaches the blue "valley" around the 11,000-step/day region across the age spectrum. This visual plateau is consistent with the statistical finding of a broad low-expenditure zone, suggesting that the fitted association—not a demonstrated cost-saving effect—plateaus around this region across age groups in the additive model. Conversely, for the middle-aged population (front of the axis), the expenditure surface remains relatively flat. This lack of variation in visualizations aligns with the lower explained variance (22.7%) observed in the statistical analysis, suggesting that, for younger cohorts, healthcare costs are less sensitive to physical activity volume and more likely to be driven by other unmeasured determinants.

### 3.5 Sensitivity analyses

**Fig. 5:**
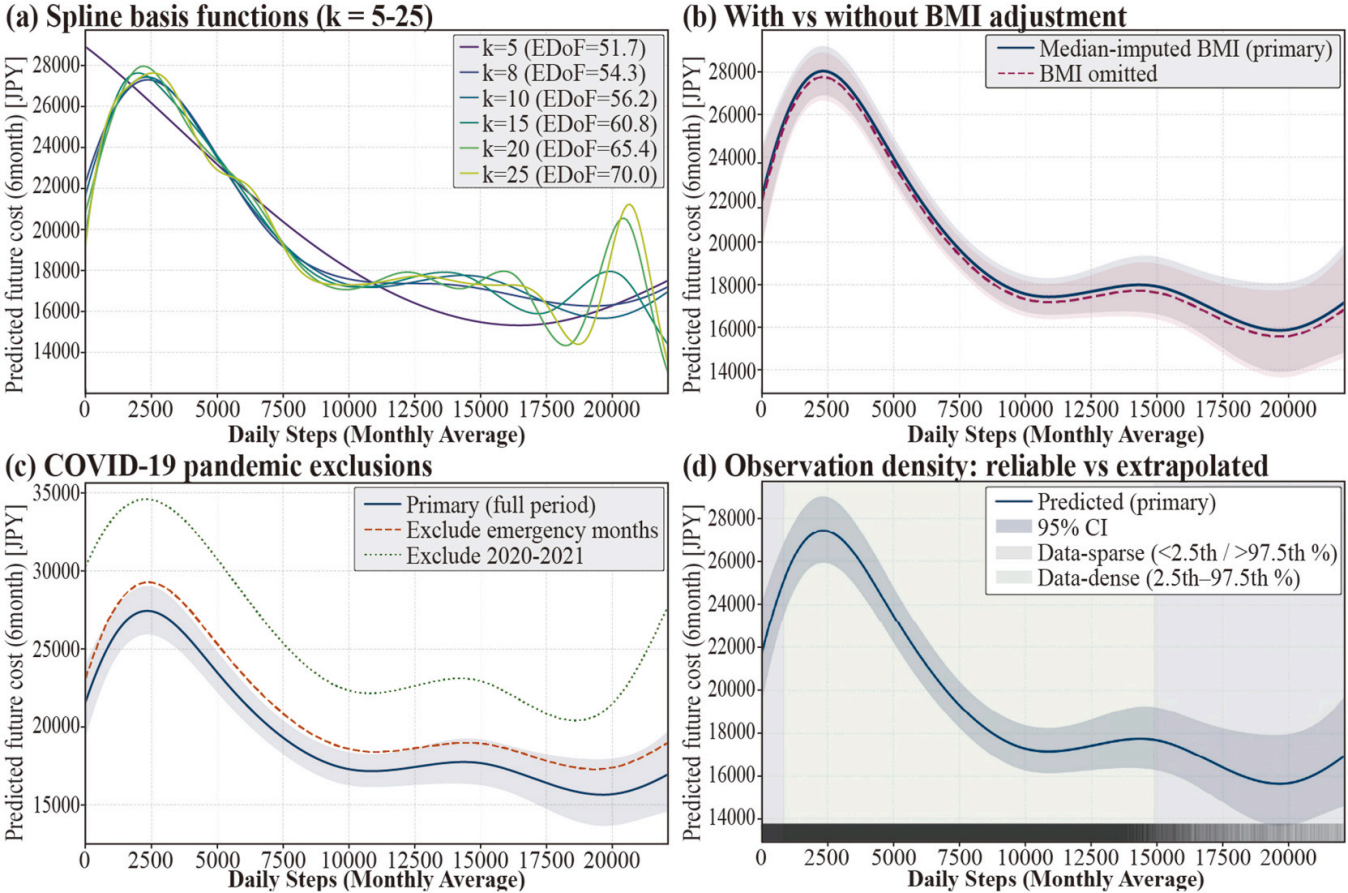
**Sensitivity analyses of the predicted step–expenditure curve.** (a) Step smooth re-estimated with the number of spline basis functions varied from 5 to 25; curves coincide within the data-dense range and diverge only in the sparse high-step region. (b) Curve with median-imputed BMI (primary) versus with BMI omitted; the two coincide, confirming insensitivity of the step smooth to missing-BMI handling. (c) Curve under COVID-19 period exclusions (full period; excluding state-of-emergency months; excluding calendar years 2020–2021). (d) Primary curve annotated by observation density, distinguishing the data-dense range (2.5th–97.5th percentile, 845–14,906 steps/day) from sparse, extrapolated ranges. Across all panels, the local nadir near 10,000–11,000 steps/day was stable, whereas the high-step global minimum was not.

The local nadir of the step–expenditure curve and the overall conclusions were broadly stable across the model-specification sensitivity analyses conducted here (Table 7, Table 8; Fig. 5). First, comparing nested specifications fitted on the same analytic observations, the intercept-only null model explained none of the deviance, a fully linear model explained 5.2%, a model with smooth terms for all covariates but a linear step term explained 32.5%, and the primary GAM with a smooth step term explained 32.6% (Table 7). Most of the explanatory power, therefore, was derived from the non-linear covariate terms—chiefly baseline medical expenditure—rather than from the step term itself; nonetheless, replacing the linear step term with a smooth significantly improved model fit (ΔAIC = 167.4), indicating that the non-linear step–expenditure association was statistically supported even though its marginal contribution to explained deviance was small.

**Table 7:** Model comparison on the primary analytic sample.

Model	Step term	Other covariates	Explained deviance	AIC	EDoF
Null (intercept only)	—	—	0.000	—	—
Fully linear	linear	linear	0.0515	1,920,941	8.0
Step-linear	linear	smooth	0.3252	1,805,725	49.1
Full GAM (primary)	smooth	smooth	0.3255	1,805,557	56.2

**Note:** Primary analytic sample (n = 338,706 person-months); 6-month cumulative outcome. ΔAIC (full GAM vs step-linear) = 167.4; Δ explained deviance = 0.0003. The non-linear step term improved fit significantly but contributed little additional explained deviance, most of which arose from the smooth baseline-expenditure and age terms.

**Table 8:** Sensitivity of the step smooth to n_splines.

n_splines	Model EDoF	AIC	GCV	Explained deviance	Local nadir	Global min
5	51.7	1,805,635	12.100	0.3254	16,450*	16,450
8	54.3	1,805,560	12.098	0.3255	19,229*	19,229
10	56.2	1,805,557	12.098	0.3255	10,893	19,674
15	60.8	1,805,547	12.097	0.3256	10,448	22,119
20	65.4	1,805,544	12.097	0.3256	9892	22,119
25	70.0	1,805,538	12.097	0.3256	9892	22,119

**Note:** Primary model uses n_splines = 10. Local nadir and global minimum are in steps/day. *At n_splines = 5 and 8 the step smooth was too rigid to resolve the data-dense local nadir, so the single minimum fell in the sparse high-step region. Explained deviance was stable across n_splines, and the fitted curves were similar within the data-dense range; these findings argue against substantial sensitivity to the chosen basis dimension in the primary interpretive range. The smoothing penalty selected by GCV controls curve flexibility, although the sparse high-step tail remained sensitive to basis choice.

Second, varying the number of spline basis functions for the step smooth from 5 to 25 produced near-identical curves within the data-dense range, and the explained deviance was stable (0.325–0.326; Table 8, Fig. 5a). The local nadir within the observed range was consistent for models with sufficient flexibility (approximately 9900–10,900 steps/day for n_splines ≥ 10), whereas the position of the global minimum in the sparse, high-step region (>15,000 steps/day) shifted markedly with n_splines (16,450–22,119 steps/day). This instability confirms that the high-step global minimum is an extrapolation artifact rather than a robust target, consistent with the wide confidence intervals and low observation density in that range (Fig. 5d).

Third, omitting the BMI smooth term—addressing the possibility that BMI lies on the causal pathway and acts as a mediator rather than a confounder—left the curve essentially unchanged (local nadir 10,893 steps/day both with and without BMI; Fig. 5b). Fourth, with respect to the COVID-19 period, excluding state-of-emergency months (n = 284,394) and excluding calendar years 2020–2021 (n = 212,106) shifted predicted expenditures upward in level but preserved the curve shape and the local nadir (11,004 and 10,782 steps/day, respectively; Fig. 5c). Notably, the elevated predicted expenditures at very low step counts persisted—indeed became more pronounced—when the pandemic period was removed, indicating that this feature is not an artifact of pandemic-era behavior. Finally, given that BMI was missing for 47.8% of analytic person-months, we assessed sensitivity to BMI handling by comparing the median-imputed primary model with a specification that omitted the BMI smooth entirely. The local nadir was 10,893 steps/day in both (Fig. 5b). Because removing BMI from the model altogether left the step curve essentially unchanged, the estimated step–expenditure association appears unlikely to be materially driven by median imputation of BMI. However, because BMI was self-reported and missing for a substantial proportion of person-months, residual uncertainty about BMI measurement and missingness remains.

## 4 Discussion

### 4.1 Non-monotonic association between daily steps and healthcare expenditures

The primary finding of this study is the identification of a distinct non-linear, non-monotonic association between daily step counts and future healthcare expenditures in a super-aged society. By employing GAM on a large-scale linked dataset, this study showed that the adjusted association between device-recorded steps and predicted expenditures does not follow a simple linear monotonic decrease. Specifically, predicted expenditure peaked at approximately 2500 steps/day at the sedentary level, followed by a steep decline in the lower step-count range. This finding is consistent with prior economic studies, which indicate that the highest healthcare costs are concentrated among sedentary or inactive populations (Kang & Xiang, 2017; Tsuji et al., 2003). Below approximately 2500 steps/day the fitted curve turned downward, so that the very lowest recorded activity was associated with lower—not higher—predicted expenditure. We caution against a substantive reading of this left-tail feature: because no day-level exclusion of low step counts was applied, monthly-mean step counts in this range may partly reflect intermittent device carriage or app malfunction (Althoff et al., 2017) rather than genuinely near-zero ambulation. The available data do not distinguish these mechanisms from true low activity at the person-month level; therefore, the lowest-step person-months are a heterogeneous group that does not cleanly correspond to the frailest individuals. This behavior, together with the sparse, extrapolated region below the 2.5th percentile (Fig. 5d), is best regarded as a measurement-and-density feature rather than a protective effect of inactivity; we therefore confine interpretation to the data-dense, monotonically declining range between approximately 2500 and 11,000 steps/day. Interpreted through a health capital and health production lens (Grossman, 1972), this pattern is compatible with the hypothesis that expenditure differences are larger at lower activity levels and smaller near the plateau. However, the present observational analysis cannot determine whether increasing steps from a sedentary baseline would produce equivalent reductions in medical care demand.

In the primary model, the fitted curve had a local nadir at approximately 11,000 steps/day. Continuous mHealth step-count data allowed the shape of the adjusted association to be examined without imposing a linear step term. The local nadir should not be interpreted as a precise threshold or as evidence that increasing an individual's step count to this level would reduce healthcare expenditures. Rather, it describes a relatively low and flat portion of the fitted curve in this selected cohort. The predicted cumulative expenditure at the local nadir was 17,170 [95% CI: 16,174–18,229] JPY. Although this region is broadly near the plateau reported in some epidemiological studies of steps and clinical outcomes (del Pozo Cruz, Ahmadi, Lee, et al., 2022; del Pozo Cruz, Ahmadi, Naismith, et al., 2022; Paluch et al., 2022), those studies evaluated different outcomes and do not validate an expenditure target.

The model also depicted a "global minimum" at the extreme activity level of approximately 20,000 steps/day. However, given the scarcity of users consistently achieving this level, this likely reflects selection bias (healthy user effect) or model overfitting rather than a replicable physiological benefit. Previous systematic reviews have suggested that excessive walking (beyond ∼12,000 steps/day) may yield diminishing returns or even increase the risk of musculoskeletal disorders (Duijvestijn et al., 2023). Our sensitivity analyses, however, indicate that the high-step behavior of the curve is not robust: the location of this apparent global minimum shifted between approximately 16,000 and 22,000 steps/day as the flexibility of the spline was varied, and it lay within a data-sparse region in which the curve is effectively extrapolated (Section 3.5; Fig. 5a and d). We therefore interpret both the apparent global minimum at approximately 20,000 steps/day and the slight intermediate fluctuation around 13,000–15,000 steps/day as artifacts of extrapolation, model flexibility, and selection (healthy-user effect) among the few users sustaining such volumes, rather than as a replicable physiological optimum. Accordingly, we neither attribute the intermediate fluctuation to a specific mechanism such as musculoskeletal overhead nor treat approximately 20,000 steps/day as an expenditure-minimizing target, and we confine interpretation to the lower, data-dense range.

Furthermore, the 6-month accumulation window adopted here should be understood as an analytic choice balancing interpretability and estimation stability across the windows examined, not as an estimate of the latency at which physical activity causally affects expenditures. This descriptive pattern is consistent with the idea that physiological benefits require an accumulation period to translate into reduced medical utilization, implying that policy interventions must be designed with a medium-term perspective (Ding et al., 2016; Saito & Podestà, 2024). A window of only a few months may nonetheless be insufficient to capture the gradual processes—operating over years—through which sustained activity is thought to influence chronic-disease risk and utilization; conversely, a short lag heightens vulnerability to reverse causation, whereby an as-yet-undiagnosed condition simultaneously depresses step counts and is followed by higher expenditures. Adjustment for baseline expenditures attenuates, but does not remove, this concern, and we therefore treat the temporal pattern as descriptive rather than as evidence of a specific causal delay.

### 4.2 Theoretical and practical implications

From a practical perspective, the non-uniform shape of the cost curve implies that the cross-sectional expenditure gradient associated with a given difference in steps depends on the baseline activity level at which that difference is located: an increment from a sedentary baseline maps onto a steeper portion of the curve than the same increment near the plateau. In principle, pairing causally identified intervention effects on walking (e.g., Aldred et al., 2019; He et al., 2021; Kato, 2025, 2026; Kato et al., 2025; Nakajima & Kato, 2025) with this curve could support exploratory Social Impact Assessments comparing relative efficiency across target populations. Any such estimates would be illustrative only: they are observationally derived, and aggregating geometric-mean predictions would understate budgetary totals given the heavy-tailed cost distribution, so absolute fiscal projections would require re-transformation (e.g., smearing estimators).

Ultimately, this SIA framework illustrates how the Grossman health capital model (Grossman, 1972), introduced at the outset of this study, might be extended conceptually from an individual behavioral concept toward population-level considerations, while the present observational design cannot itself support a fiscal strategy. While Grossman conceptualized time spent on physical activity as an individual's investment to produce health, our findings suggest that features of the urban environment may also serve as the ‘infrastructure’ shaping the efficiency of this health production process (McLeroy et al., 1988; Sallis et al., 2016). Such environmental considerations, however, are most usefully framed as one component of the multi-level, whole-of-system approach articulated in the WHO Global Action Plan on Physical Activity 2018–2030 (WHO, 2018), which situates supportive environments alongside societal, individual, and systems-level strategies rather than as a substitute for them. By leveraging urban design to create environments that support active lifestyles, municipalities may contribute to—rather than replace—broader public-health efforts (Mouratidis, 2024). To the extent that strategies shift the population distribution towards higher physical activity levels, they may support societal health capital and could contribute to the financial sustainability of healthcare systems (Saito & Podestà, 2024).

Regarding generalizability, these results are specific to a selected cohort of long-term app users in a single prefecture and do not support population-level or cross-national targets. At most, the non-linear association observed in Osaka may help generate hypotheses for other super-aged settings; testing them would require replication in different populations and insurance systems before any policy translation. If future causal evaluations confirm that walkability interventions increase sustained physical activity and reduce healthcare utilization, investing in walkability may carry economic as well as health rationales. Whether it constitutes a cost-effective strategy for mitigating future financial strain, however, requires direct evaluation that the present observational design cannot provide. Any fiscal estimates based on the present curve should therefore be regarded as exploratory. Although the non-linear step term significantly improved model fit, it accounted for only a marginal share of the explained deviance (Δ pseudo-R^2^ ≈ 0.0003; Table 7), the great majority of which was attributable to baseline medical expenditure. The fitted curve therefore characterizes an associational shape rather than providing a precise predictive model of individual expenditure or a direct basis for fiscal strategy.

### 4.3 Research limitations

Several limitations of this study must be acknowledged. First, potential selection bias is inherent in the use of mHealth applications. The study population consisted of A-Smile users who may have had greater health awareness and digital literacy than the general population, and inclusion in the analytic cohort additionally required sustained app use, successful linkage to claims data, and enrollment in an eligible insurance scheme. The slightly higher mean daily step count among older than middle-aged participants may partly reflect differential health and digital selection rather than an age-related difference in the underlying population. The older-adult sample was also younger than the corresponding Osaka population benchmark, consistent with the underrepresentation of the oldest-old. A-Smile users likely have higher health and digital literacy than the general population and may have higher socioeconomic status. This "healthy user effect" suggests that our sample may represent a relatively healthier segment of society, thereby limiting the generalizability of both the overall and age-specific step–expenditure curves and their local nadirs to all middle-aged and older residents of Osaka. Because health-related selection is plausibly stronger at higher step counts—healthier individuals both walk more and sustain app use—selection is expected to bias the estimated curve in identifiable directions: absolute expenditure levels are biased downward relative to the target population, while the apparent expenditure gradient across the data-dense range may overstate any causal step–expenditure relationship. However, because healthcare expenditures were not linkable for excluded users and the app cohort was not probability-sampled, this study could not quantify the magnitude of selection bias in the fitted step–expenditure curve. The local nadir near 11,000 steps should accordingly be read as the level at which this selected, relatively healthy subpopulation concentrates, not as a level whose attainment would by itself lower an individual's expenditures.

Second, despite adjusting for key covariates such as age, gender, BMI, and baseline medical expenditures, this study's analysis was limited by the absence of other potential confounders that are often not captured in administrative claims data. Specifically, this study could not account for socioeconomic factors (e.g., income, education), lifestyle habits other than walking (e.g., smoking, alcohol consumption, dietary quality), or specific genetic predispositions. These unmeasured factors could independently influence both physical activity levels and healthcare utilization.

Third, the exposure assessment relied on smartphone-based step counts, which have inherent limitations. While commercially available smartphones have demonstrated acceptable validity for step counting, they may not capture non-ambulatory activities such as cycling, swimming, or resistance training. Consequently, the total physical activity volume of some users may have been underestimated. Furthermore, unlike research-grade accelerometers, smartphone carriage positions were not standardized, which may have introduced measurement error. These factors, together with the inclusion of the COVID-19 period—during which population step counts fell substantially (Matsumoto et al., 2026)—and the restriction of the analytic sample to National Health Insurance and elderly-insurance enrollees (who skew older and are less likely to be in commuting-based employment), help account for the observed mean of approximately 5000 steps/day, which is lower than national or metropolitan benchmarks. The absolute step level should therefore be interpreted as a property of this measurement context and subpopulation rather than as an estimate of underlying population activity.

Fourth, while we adjusted for COVID-19 emergency declaration months, residual confounding by seasonality and secular time trends cannot be excluded. In addition, pandemic-era reductions in step counts may have been socially patterned: users with higher socioeconomic status, greater health literacy, or greater access to telework may have reduced outings without necessarily having poorer underlying health. This mechanism could have changed the composition of low-step person-months and may partly explain the unusual behavior of the fitted curve in the lowest step-count range. Because the present models did not include explicit calendar-time or seasonal terms, future work could additionally adjust for calendar time (e.g., year–month) and seasonality (e.g., a cyclic month-of-year term) to assess whether the estimated step–expenditure curve and the plateau/threshold region are robust to time-varying confounding.

Finally, although this study used a lagged-outcome design and adjusted for past medical expenditures to mitigate reverse causality, its observational design precludes definitive causal inference. The 6-month baseline-expenditure adjustment removes confounding from health problems that have already become manifest as reimbursed care, but it cannot capture subclinical conditions that depress physical activity before they generate any claim; such conditions could independently raise subsequent expenditures and thereby steepen the apparent step–expenditure association. Epidemiological studies of activity and clinical outcomes commonly impose 1- to 2-year washout periods to limit reverse causation. In the present cohort, a baseline window of this length could be constructed only for the subset of later index months with at least 12 months of prior app-linked data, which would substantially reduce the analytic sample and shift its composition; we therefore retain the 6-month baseline as the primary specification and identify a longer-baseline analysis as a priority for future work as the A-Smile–claims linkage matures. Residual reverse causation may affect the steepness of the fitted gradient, absolute expenditure levels, and potentially the apparent location of lower-expenditure regions. Although the local nadir lies within the data-dense mid-range (∼11,000 steps/day) rather than in the sparse low-activity tail, it should still be interpreted as a descriptive feature of this selected cohort rather than as robust evidence of a causal threshold. Our maximum follow-up of approximately four years likewise constrains inference about the longer-term accumulation of benefits. These findings should therefore be read as associational; confirmation will require replication with longer baseline and follow-up windows—ideally a washout of at least 12 months—which the maturing A-Smile–claims linkage will increasingly permit.

Beyond these design issues, the explanatory contribution of daily steps to future expenditure was modest. Although the non-linear step–expenditure association was statistically robust, daily steps accounted for only a small fraction of the variation in future expenditure beyond baseline medical costs (Table 7). The practical value of step counts for forecasting individual expenditure is therefore limited, and our findings are best read as characterizing a population-level associational shape rather than as a basis for individual prediction.

## 5 Conclusion

This study provides empirical evidence elucidating the non-linear relationship between mHealth-measured daily steps and future healthcare expenditures in a super-aged society. By leveraging large-scale mHealth data and GAM, this study challenges the traditional linear assumption and characterizes a relatively flat, lower-expenditure range beginning around 10,000–11,000 steps/day, where the marginal reduction in predicted expenditures diminishes. Because the analysis is observational and confined to a selected cohort, this range is best understood as an associational benchmark for generating hypotheses rather than as a causal target whose attainment would lower a given individual's expenditures. The findings may inform future studies evaluating physical-activity interventions, environmental supports, and broader systems-level strategies, but they do not establish that any specific intervention would reduce healthcare expenditures. As the global population ages, this case from Osaka may help generate hypotheses for other super-aged settings; replication with longer baseline and follow-up windows, more representative samples, and causally identified intervention designs will be required before any policy prescription is warranted.

## Ethical statement

The research protocol was approved by the Ethics Committee of the Graduate School of Human Life and Ecology, Osaka Metropolitan University (No.24-42). Informed consent was obtained electronically from all users, and continued app use could be declined at any time through the smartphone application, in accordance with the Declaration of Helsinki (Osaka Prefecture, 2025b, 2025c). Furthermore, this approach is consistent with Japan's Next-Generation Medical Infrastructure Act (Cabinet Office of Japan, 2025), which aims to promote the utilization of medical information through organizations accredited by the Japanese government as "Accredited Businesses for Anonymized Medical Information" (Japanese Law Translation, 2025). These accredited businesses can, with user permission, process collected information into an anonymized format that prevents personal identification and provide it to research institutions, companies, and other users. Through this process, which is under the supervision of the Japanese government, the security and ethical integrity of this research protocol were addressed (Osaka Prefecture, 2025d).

## Declaration of generative AI use

During the preparation of this work, the author used Claude (Anthropic) and ChatGPT (OpenAI) in order to improve the readability and language of the manuscript. After using this tool/service, the authors reviewed and edited the content as needed and take full responsibility for the content of the published article.

## Funding

This work was supported by 10.13039/501100008042Obayashi Foundation Research Program, Japan (grant number 2024-27-28), the 10.13039/501100012009Hitachi Global Foundation, Japan (grant number 1622), the Osaka Metropolitan University Co-Creation Research Group Formation Support Program, Japan (2026-03), and the JST COI-Next, Japan (JPMJPF2115). The opinions, findings, conclusions, or recommendations expressed in this manuscript are those of the author.

## Declaration of interests

The authors declare that they have no known competing financial interests or personal relationships that could have appeared to influence the work reported in this paper.

## Data Availability

The authors do not have permission to share data.
